# Expressed sequence tags (ESTs) from young leaves of *Metroxylon sagu*

**DOI:** 10.1007/s13205-012-0048-6

**Published:** 2012-03-06

**Authors:** Ching Ching Wee, Hairul Azman Roslan

**Affiliations:** Genetic Engineering Laboratory, Department of Molecular Biology, Faculty of Resource Science and Technology, Universiti Malaysia Sarawak, 94300 Kota Samarahan, Sarawak, Malaysia

**Keywords:** EST, cDNA, Sequencing, Metroxylon sagu, Sago palm

## Abstract

Sago palm, or *Metroxylon sagu*, is a hardy and versatile plant that is able to tolerate many stresses, biotic and abiotic, during its growth. It is one of the plants that are able to grow in waterlogged area where others could not. Apart from that sago palm is also a source of starch, contributes economically to the people and an important export for the state of Sarawak. Despite the importance of sago palm especially in the production of starch and its ability to withstand stresses, so far, not many molecular studies have been reported on sago palm. To study the characters in sago palm, transcriptome analysis was conducted where it would give a better understanding of the plant development through gene expression. Here, we report the construction of a cDNA library and preliminary expressed sequence tags analysis from the young leaves of sago palm. A total of 434 clones were sequenced with inserts ranging from 1,000 to 3,000 bps with primary and amplified titers of 8 × 10^5^ and 1.0 × 10^9^ pfu/ml, respectively. Clustering of these sequences resulted in a set of 372 tentative unigenes comprising 340 singletons and 32 contigs. The database was also annotated with BLAST2GO which showed that majority of the transcripts were involved in primary metabolism and stress tolerance.

## Introduction

Sago palm (*Metroxylon sagu* Rottb.) is a monocotyledonous plant belonging to the order Arecales, family Palmae, and subfamily Calamoideae. This plant grows well at temperature of 25 °C and above together with 70% humidity. The plant can be found around South East Asia and diffused from its native to other places due to human activity (Yen 1995). It is an important agricultural crop for the state of Sarawak, Malaysia, and having the largest growing area in the state. Sarawak is also one of the world’s biggest exporter of sago starch, exporting 44,700 tonnes of sago starch in 2007 to Japan, Taiwan, Singapore and other countries, procuring incomes of between US$3.4 million to US$10.8 million (DOA, Sarawak). Starch, accumulated in the pith of sago palm, is the major product of sago palm. It has been reported that the production of starch from sago palm is four times higher than starch derived from rice (*Oryza sativa*) (Lal 2003). Another advantage of sago palm is in its ability to grow in swamp or waterlogged area in the South East Asia and surrounding (Singhal et al. 2008), and acidic peat soil with high concentration of metals such as aluminum, iron, and manganese, to which most crops are unable to thrive (Yen 1995). Depite its adaptability towards harsh conditions and the economic importance of sago palm, reports on molecular work for this plant is very few.

An expressed sequence tag (EST) library was constructed to gain a better understanding of the molecular mechanism and gene expression during sago palm development and its ability to withstand the various stresses. The cDNA library created contained the expression pattern of a selected tissue is an effective tool for research of gene expression. The ESTs generated from specific tissues represents the presence of active mRNAs in the selected tissue and sampling conditions. The creation of an EST database has several advantages including a fast and inexpensive way to discover novel genes, rapid identification of active genes, identification of exon–intron structure, generation of information on gene expression, gene regulation and sequence diversity, comparative genomic study, serve as markers or tags for transcripts, development of markers for reference genetic map and recovery of full-length cDNAs and genomic sequences (Ho et al. 2007; Luro et al. 2008; Thanh et al. 2011; Trivedi et al. 2003; Ye et al. 2010; Zeng et al. 2010).

The creation of an EST database is an approach that could accelerate the researches of non-model and emerging species such as *Metroxylon sagu*. The EST approach is a potential resource for several follow-up studies, such as functional and comparative genomics, which has been successfully used in other model species (soy beans, *Arabidopsis*, etc.) and non-model species (oil palm, citrus, herbs, etc.). As for the palm family, extensive EST database have only been established for oil palm (*Elaeis guineensis*) (Ho et al. 2007; Jouannic et al. 2005; Low et al. 2008). Nevertheless, not many DNA sequences are available in the public databases for other Arecaceae family including *Metroxylon sagu*. In addition, EST approach is also an efficient, comparatively cheap, a powerful tool for gene discovery, to investigate transcriptomes and identify genes regulated by development and abiotic stresses (Lindqvist et al. 2006; Thanh et al. 2011; Wang et al. 2007). To the best of our knowledge, no EST study has been reported for sago palm. Therefore, here we report a preliminary ESTs analysis of young leaf samples from sago palm.

## Materials and methods

### Plant material

Young leaves of sago palm (*Metroxylon sagu*) were obtained from Universiti Malaysia Sarawak (UNIMAS) plant house. The midrib and stems of the leaves were discarded; the leaf was washed with distilled water, surface sterilized using 70% ethanol and cut into small pieces. The samples were then stored at −80 °C until required.

### Isolation of total RNA

RNA isolation was carried out according to Gasic et al. (2004) method with slight modification as followed: a 10 ml extraction buffer [2% CTAB, 2% polyvinylpyrrolidone (PVP 40), 100 mM Tris–HCl (pH 8.0), 25 mM EDTA, 2.0 M NaCl, 2% β-mercaptoethanol (added just before use)] was pre-warmed at 60 °C in a water bath. Approximately 5 g of leaf samples was ground into a fine powder in liquid nitrogen with a mortar and pestle. The powdered tissue was transferred into a 50 ml falcon tube containing extraction buffer and incubated at 60 °C for 30 min. An equal volume of chloroform:isoamyl alcohol [24:1 (v/v)] was added and immediately vortexed for 1 min. The mixture was then centrifuged at 8,000 rpm for 30 min at 4 °C. After centrifugation, the upper aqueous phase was transferred to 1.5 ml microcentrifuge tubes and re-extracted with equal volume of chloroform:isoamyl alcohol. The upper aqueous phase was transferred to new tube and one-third volume of 8 M lithium chloride solution was added to a final concentration of 2 M. The tubes were incubated overnight at −20 °C and centrifuged at 13,000 rpm for 30 min at 4 °C. The supernatant was carefully discarded and the pellet was washed with 500 μl of 70% ethanol followed by 500 μl of 80% ethanol. After the pellet was air-dried, it was re-suspended in 35 μl DEPC-treated water. Total RNA was stored at −80 °C for long-term storage. The RNA was analyzed via gel electrophoresis and spectrophotometer (Ultrospec^®^ 1100 pro, Amersham Pharmacia Biotech). Trace amount of genomic DNA was then removed by treating the total RNA with RQ1 RNase-Free DNase (Promega).

### cDNA library construction

The poly(A) mRNA from total RNA was then enriched using the Oligotex mRNA Spin-Column (Qiagen) prior to library construction. The cDNA libraries were then constructed using ZAP-cDNA synthesis kit (Stratagene) and ZAP-cDNA Gigapack^®^ III Gold packing extract (Stratagene). Double-stranded cDNA was synthesized using ZAP-cDNA synthesis kit followed by blunting the cDNA termini. *EcoR*I adapters were then ligated to both cDNA ends and the oligo(dt) linker-primer that contained the *Xho*I restriction site was digested using *Xho*I restriction enzyme to produce *Xho*I overhangs. Next, the cDNAs were size-fractionated using a drip column containing Sepharose^®^ CL-2B gel filtration medium. The fractionated cDNA was then precipitated and ligated into Uni-ZAP XR vector. The ligated product was packaged using ZAP-cDNA Gigapack^®^ III Gold packing extract (Stratagene), titered and amplified to at least 10^8^ pfu/ml or more.

### Phagemid isolation and DNA sequencing

Phagemid was excised in vivo from Uni-ZAP XR vector using ExAssist helper phage with SOLR™ strain. The bacteria was plated on Luria–Bertani plate containing 50 mg/l ampicillin and incubated overnight at 37 °C. Colonies were randomly picked and the phagemid was isolated using GeneJET™ Plasmid Miniprep kit (Fermentas). The phagemid were then PCR-amplified using universal T7 (5′-TAATACGACTCACTATAGGG-3′) and T3 (5′-ATTAACCCTCACTAAAGGGA-3′) primers to determine the insert size. The PCR reaction mixture include 6.5 μl Go*Taq*^®^ Green Master Mix (Promega), 0.5 μl 10 μM T7 primer, 0.5 μl 10 μM T3 primer, 9.5 μl nuclease-free water and 0.5 μl bacteria culture. The mixture was amplified according to the following cycles: 1 cycle of 94 °C for 3 min; 35 cycles of 94 °C for 30 s, 50 °C for 30 s, 72 °C for 1 min; and 1 cycle of 72 °C for 5 min. DNA sequencing was carried out on the phagemid by a commercial company, First BASE Laboratories Sdn Bhd (Selangor, Malaysia).

### ESTs processing, contig assembly and sequence analysis

Prior to sequence analysis, the sequences were processed by trimming the vector sequences using the software EGassembler (Masoudi-Nejad et al. 2006). The EST sequence assembly and consensus generation were also done using the same software (Masoudi-Nejad et al. 2006). To identify putative genes, the resulting tentative unique genes (TUGs) determinants were compared against the National Center for Biotechnology Information (NCBI) non-redundant protein database using BLASTx program with *e* value threshold 10^−3^. BLASTx results with mean similarity equal to or less than 60% were treated as ‘low homology while those with mean similarity of more than 60% were treated as ‘high homology’. Meanwhile the ESTs without matches were classified as ‘no matches’. The ESTs with hits from the BLASTx analysis were then mapped to the Gene Ontology (GO) using the BLAST2GO program (Conesa et al. 2005) and categorized according to molecular functions, biological processes and cellular components. All sequences were deposited into NCBI dbEST GenBank with accession numbers JK731189-JK731342 and JK731189-JK731600.

## Results

### cDNA library construction

**Fig. 1:**
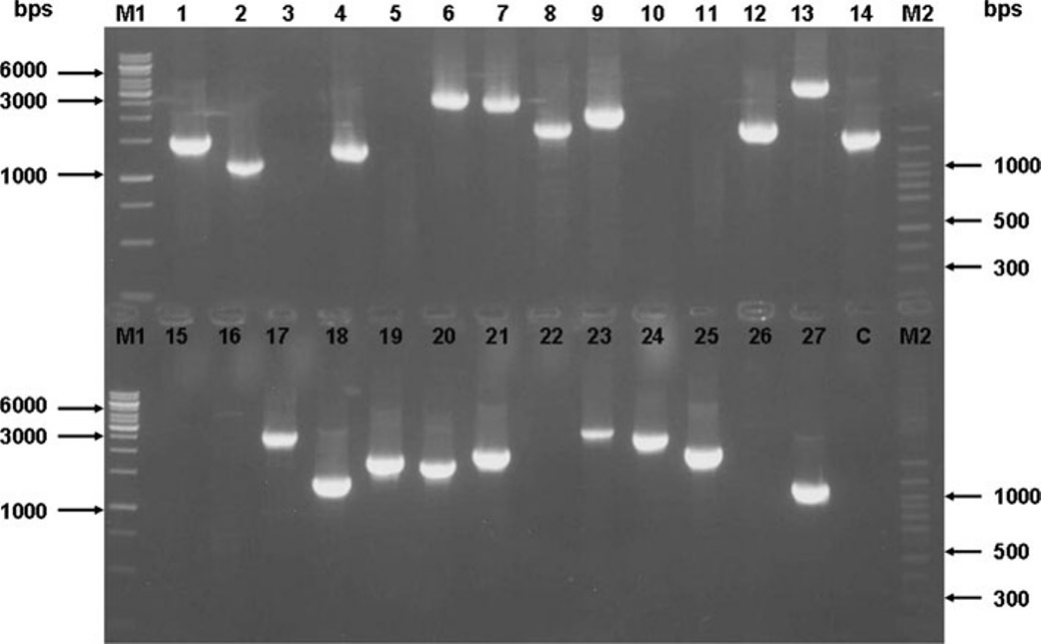
Agarose gel (1.5%) electrophoresis of colony PCR products from the sago palm young leaf EST library. T7 and T3 primers were used to amplify the plasmid. PCR results with amplicons indicate plasmids containing insert. *M1* 1 kb ladder (Fermentas), *M2* Forever 100 bp DNA ladder (Seegene), *lanes 1–27* colony PCR of samples, *C* negative control

**Fig. 2:**
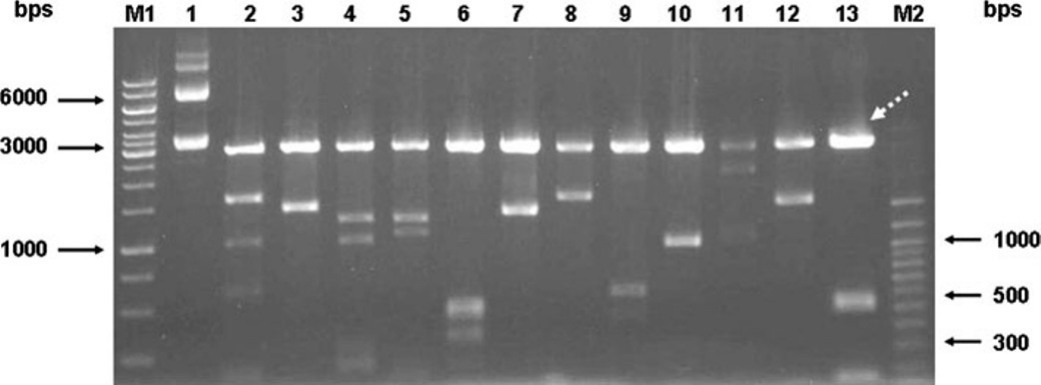
Agarose gel (1.0%) electrophoresis of plasmid digested with *Xho*I and *EcoR*I restriction enzymes. *Lane 1* undigested plasmid, *lanes 2–13* plasmids digested with *Xho*I and *EcoR*I restriction enzymes. *Dashed arrow* indicates a 3 kb representing a linearized vector fragment without insert. *M1* 1 kb ladder (Fermentas), *M2* Forever 100 bp DNA ladder (Seegene)

The cDNA library was constructed from samples derived from young leaf. The primary titer of the library constructed using commercial kit was determined to be 8 × 10^5^ pfu/ml with insertion efficiency of 99%. The library was then amplified, producing an amplified titer of 1.0 × 10^9^ pfu/ml. From the libraries, colonies containing the cDNA clones were randomly isolated and amplified with T7 and T3 primers to determine the recombinant efficiency and insert size before being sent for sequencing. From the results, the insert size for the library constructed using commercial kit ranged from 1 to 3 kb (Fig. 1). To further verify the insert size of each clone, restriction enzyme digestion was performed with *Xho*I and *EcoR*I (Fig. 2).

### EST clustering and assembly

Initially, a total of 434 clones were randomly selected and sequenced. However, 22 clones failed to be sequenced and the remaining 412 clones were analyzed. By using the EGassembler online server, low quality sequences were removed or trimmed by vector trimming, repeat and organelle masking. After that the sequences with overlap percentage of identity cutoff *N* > 65 were clustered and assembled. As a result, the sequence analysis yielded 372 tentative unique genes (TUGs) sequences that consist of 32 contigs and 340 singletons. This EST library has a redundancy of ~17%, and GC level of 45.02% (Table 1).

**Table 1:** Summary of total EST sequencing

EST	No.	%
Clean ESTs for assembly	412	
No. of consensi	32	
No. ESTs within consensus	72	17
No. of singletons	340	83
Unique sequences	372	90

### Functional annotation of EST sequences

The ESTs were analyzed using BLASTx program and a summarized distribution of the ESTs is presented in Table 2. From the analysis, approximately 86.56% of the TUGs were highly homologous to known proteins whereas 8.06% of the TUGs had low homology to known proteins. A total of 20 TUGs do not match any proteins in the database and therefore may have the potential to have novel function in sago palm.

**Table 2:** Distribution of ESTs according to the top BLASTx search results

BLASTx search results	No. of TUG (%)	No. of contigs (%)	No. of singletons (%)
High homology	322 (86.56)	29 (90.63)	293 (86.18)
Low homology	30 (8.06)	1 (3.13)	29 (8.53)
No match	20 (5.38)	2 (6.23)	18 (5.29)
Total	372 (100.00)	32 (100.00)	340 (100.00)

High homology = mean similarity ≥60%

Low homology = mean similarity <60%

**Fig. 3:**
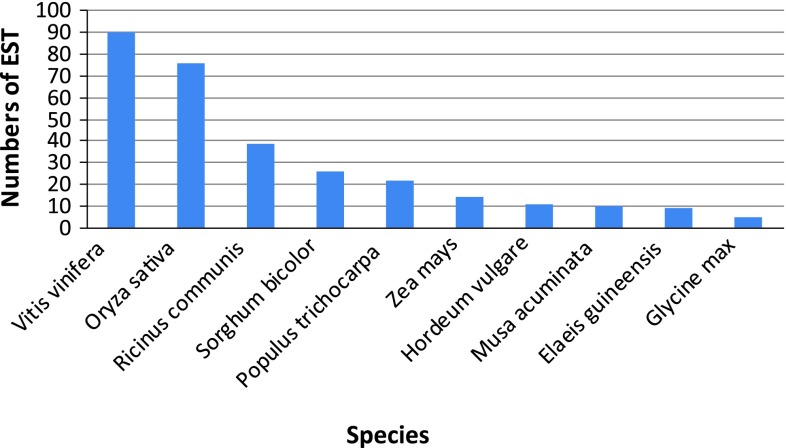
The ten most frequently matched plants using the BLASTx (BLAST2Go software)

Figure 3 shows the plant species with the highest frequency of ESTs hits with sago palm EST library that covered 81.18% of 372 ESTs. From these, six plant species (41.94%) were monocotyledons (*Oryza sativa*, *Sorghum bicolor*, *Zea mays*, *Hordeum vulgare*, *Musa acuminate* and *Elaeis guineensis*) whereas remaining four (39.24%) were dicotyledons (*Vitis vinifera*, *Ricinus communis*, *Sorghum bicolor* and *Populus trichocarpa*). Surprisingly, the BLASTx results showed almost equal distribution of sago palm (monocot) sequences in both monocot and dicot. This may be due to some of the plants database having not been fully constructed. From the BLAST2Go program analysis, the most abundant gene in the sago palm EST (Table 3) was the heat shock protein 70 and its sub-members (total of 7 ESTs). This was followed by catalase, beta-glucosidase, plastidic aldolase and cytosolic ascorbate peroxidase, each constituted 3 ESTs.

**Table 3:** BLAST2Go determination of the identity of clusters with more than 2 ESTs

Rank	Identity of cluster	Number of ESTs	*E* value	Mean similarity (%)
1	Heat shock protein 70	4	0	96
2	Catalase	3	6.8964E−141	86
3	Beta-glucosidase	3	1.75664E−99	81
4	Plastidic aldolase	3	5.12984E−158	92
5	Heat shock protein 70	3	1.23247E−137	95
6	Cytosolic ascorbate peroxidase	3	6.5858E−109	93

**Fig. 4:**
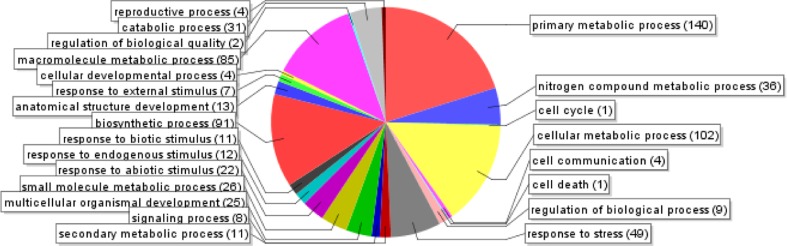
GO-annotation classification of *Metroxylon sagu* ESTs by putative biological processes (level 3)

**Fig. 5:**
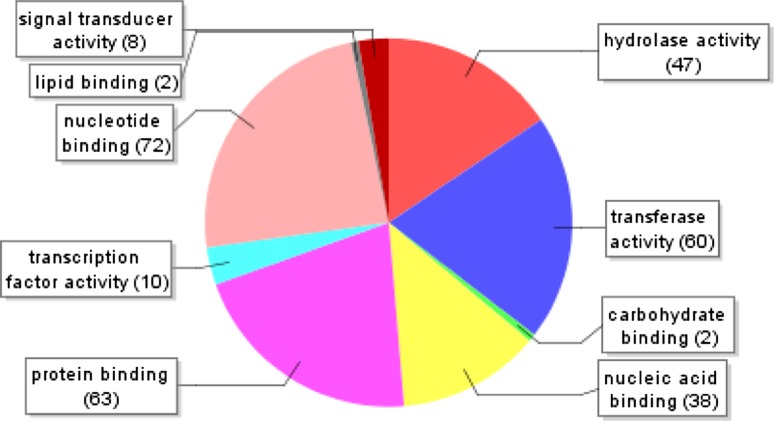
GO-annotation classification of *Metroxylon sagu* ESTs by putative molecular function (level 3)

**Fig. 6:**
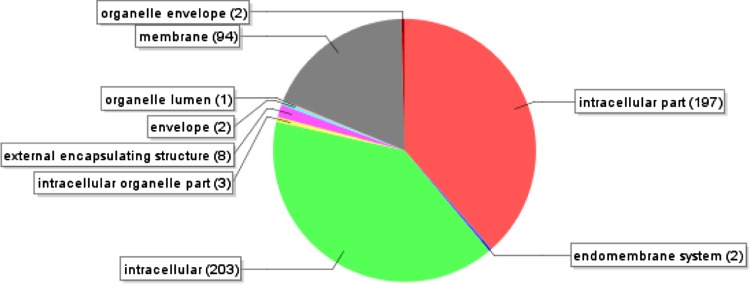
GO-annotation classification of *Metroxylon sagu* ESTs by putative cellular component (level 4)

From the annotated sequences, a total of 1,710 gene ontology (GO) terms were assigned. They were distributed in three main GO categories: biological process (2,156), molecular function (960) and cellular component (2,384). The total number under these three categories was higher than 1,710 because some GO terms was mapped to one or more GO categories. A total of 694 assignments were designated under the category of biological process (level 3) with 140 in primary metabolism (20%), 102 in cellular metabolism (14%), 85 in macromolecule metabolism (12%), and 91 in biosynthesis (13%) (Fig. 4). A total of 302 TUGs were also mapped to the molecular function (level 3) (Fig. 5), with approximately 177 (58%) in binding and the other involve in catalytic activities. Lastly, 512 assignments were assigned for cellular components (level 4) (Fig. 6) and they were divided into extracellular, intracellular and membrane. Vast majority of them were under intracellular (406, 79%) while 96 assignments were assigned to membrane and 1% were assigned to extracellular.

## Discussion

Gene expression profile of young leaves during development was studied via the development and analysis of expressed sequence tags. The sago palm young leaf EST library produced large inserts between 1 and 3 kb and the redundancy was low, indicating that many expressed gene transcripts still to be captured.

Functional categorization of the ESTs under the biological process and primary metabolites represent the highest percentages of cDNA transcripts that include protein/amino acid, carbohydrate and nucleic acid metabolism (Iturriaga et al. 2006). Glucose is one of the most important carbohydrates that are synthesized in plants through photosynthesis and stored in either starch or lipid forms. Catabolism of carbohydrate through glycolysis and aerobic metabolism of glucose provides energy in the form of ATP for the survival of organism. In this study, enzymes involved in the glycolysis pathway were detected that includes glucose-6-phosphate dehydrogenase, glyceraldehyde-3-phosphate dehydrogenase, fructose-bisphosphate aldolase and phosphoglycerate kinase. On the other hand, several transcripts that are important for the synthesis of the first fully formed purine nucleotide, inosine monophosphate (IMP) in nucleic acid metabolism was detected in ESTs database such as glutamine synthase, glycine-rich protein and aspartate aminotransferase.

The fifth most abundant functional category is the stress tolerance (49 ESTs). Reactive oxygen species (ROS) plays several roles in apoptosis, hormone/cell signaling and stress response (Jin et al. 2006). During stress, ROS levels will increase and cause significant damage to cell structure. However, the adverse effects of ROS can be overcome through the activity of enzymes, such as superoxide dismutases (SODs), catalases, lactoperoxidases, glutathione peroxidases and peroxiredoxins. Analysis of the ESTs reveals the presence of several enzymes related to the defense against ROS damage such as the glutathione *S*-transferase, peroxisome-like proteins, among other are the glycine-rich protein, catalase and alcohol dehydrogenase class III. Glutathione *S*-transferase and ascorbate peroxidase are antioxidants that protect the plants by detoxifying ROS generated during biotic or abiotic stress (Soranzo et al. 2004) while catalase degrades the ROS to water and oxygen. Lertwattanasakul et al. (2009) reported that Adh3 might play a crucial role in the control of NADH/NAD(+) balance in the mitochondria and eventually prevent the accumulation of ROS. In addition, beta-glucosidase plays a role in producing defense compounds when plants encounter stress (Matsushima et al. 2003).

Apart from the harsh growing conditions of sago palm, young shoot may also encounter stresses from pathogen attack, wound, application of chemicals, ozone and ultraviolet rays. Therefore, plants protect themselves through the synthesis of defense-related proteins. Several defense-related proteins were found among the ESTs including cysteine protease, phenylalanine ammonia-lyase, 1-aminocyclopropane-1-carboxylate (ACC) oxidase, Rubisco subunit binding-protein beta subunit, cytosolic ascorbate peroxidase, thylakoid-bound ascorbate peroxidase and class I chitinase. Phenylalanine ammonia-lyase activity provides precursors for the biosynthesis of lignin (Hahlbrock and Scheel 1989) and salicylic acid (SA) (Yalpani et al. 1993). Meanwhile, SA has been shown to be essential for plant systemic acquired resistance (SAR) (Vernooij et al. 1994) whereas lignification is a defense response of plants against pathogen attack (Ride 1983). Others such as cysteine protease have been shown to play a role as antifungal protein in millet seeds (Joshi et al. 1998). Apart from that ACC oxidase, Rubisco, cysteine protease, ascorbate peroxidase and chitinase have been reported to be expressed during ozone exposure (Heath 2008).

The main role of leaves is to capture light energy and produce sugars through photosynthesis. Thus, several transcripts involved in photosynthesis were detected such as ferredoxin-NADP^+^ reductase, chloroplast ribulose-bisphosphate carboxylase oxygenase small subunit, oxygen-evolving enhancer protein, chlorophyll *a*, *b*-binding apoprotein CP26 precursor, phosphoglycerate kinase, glyceraldehyde-3-phosphate dehydrogenase, protochlorophyllide reductase B, PGR5-like protein 1A and chloroplast fructose bisphosphate.

Photosynthesis, respiration and transpiration are the three major functions that drive plant growth and development. Apart from that, genes essential to plant growth and development, among other are the *S*-adenosylmethionine decarboxylase, class I chitinase, ubiquitin-specific protease, isoleucyl-tRNA synthetase, homeodomain leucine zipper protein, glycogen synthase kinase-3, DNA-binding protein WRKY2 and anthranilate synthase component I family had been detected in the EST database. In addition, plant hormones are also important in regulating plant growth and development. A number of plant hormone-related genes were found among the ESTs. These include ethylene responsive/signal transcription factor, auxin-regulated gene, auxin-response factor 9, auxin-induced protein and auxin efflux carrier family protein. Several reasons can be attributed to the activation of these hormone-related genes, such as environment stresses and wounding. Activation of hormone signaling in turn regulates the plant response to stress by affecting subsequent gene expression and protein synthesis (Carrera and Prat 1988; Dammann et al. 1997; Ershova et al. 1991; Vojnikov and Ivanova 1988; Zhyrmunskaja et al. 1989). Ethylene response factor that was detected is a positive regulator that binds to the promoter elements of ethylene-regulated gene (Chang and Shockey 1999) thus preventing over-expression of ethylene that have been shown to cause growth retardation and flowering delay in *Arabidopsis* (Achard et al. 2007). In contrast, gibberellin (GA) plays an important role in stress protection by promoting growth (Jackson 2008). The 14-3-3 protein is a stress-signaling component that regulates primary metabolism, ion transport, gene expression and may be used for improving stress tolerance (Yan et al. 2004). Gh3 family genes are proteins that response after auxin treatments (Guilfoyle 1999) meanwhile auxin-response factors (ARF) are transcriptional activators and repressors that bind to the promoters of auxin-response gene (Tiwari et al. 2003).

Lastly, around 5.38% of the analyzed sequences did not match with any sequence in the NCBI database. These unknown and unclassified cDNAs could in turn be novel genes for sago palm.

## Conclusion

Finally, we have shown that the EST database generated have been useful in determining the mRNA species present in sago palm leaf tissue. The work presented here served as a basis for further molecular analysis for sago palm and also indicated that sago palm is a useful plant to study the defense-related and stress-related proteins apart from studying the primary metabolism in newly developed shoots. Nevertheless, this finding from the work is a limited and serves as a preliminary assessment of the transcripts in a selected sago palm tissue and currently efforts are underway to increase the number of ESTs derived from these tissues.
